# Structural basis of IL-23 antagonism by an Alphabody protein scaffold

**DOI:** 10.1038/ncomms6237

**Published:** 2014-10-30

**Authors:** Johan Desmet, Kenneth Verstraete, Yehudi Bloch, Eric Lorent, Yurong Wen, Bart Devreese, Karen Vandenbroucke, Stefan Loverix, Thore Hettmann, Sabrina Deroo, Klaartje Somers, Paula Henderikx, Ignace Lasters, Savvas N. Savvides

**Affiliations:** 1 COMPLIX N.V., Technology Park 4, 9052 Ghent, Belgium; 2 Unit for Structural Biology, Laboratory for Protein Biochemistry and Biomolecular Engineering (L-ProBE), Department of Biochemistry and Microbiology, Ghent University, K.L. Ledeganckstraat 35, 9000 Ghent, Belgium; 3 Unit for Biological Mass spectrometry and Proteomics, Laboratory for Protein Biochemistry and Biomolecular Engineering (L-ProBE), Department of Biochemistry and Microbiology, Ghent University, K.L. Ledeganckstraat 35, 9000 Ghent, Belgium; 4 These authors contributed equally to this work

## Abstract

Protein scaffolds can provide a promising alternative to antibodies for various biomedical and biotechnological applications, including therapeutics. Here we describe the design and development of the Alphabody, a protein scaffold featuring a single-chain antiparallel triple-helix coiled-coil fold. We report affinity-matured Alphabodies with favourable physicochemical properties that can specifically neutralize human interleukin (IL)-23, a pivotal therapeutic target in autoimmune inflammatory diseases such as psoriasis and multiple sclerosis. The crystal structure of human IL-23 in complex with an affinity-matured Alphabody reveals how the variable interhelical groove of the scaffold uniquely targets a large epitope on the p19 subunit of IL-23 to harness fully the hydrophobic and hydrogen-bonding potential of tryptophan and tyrosine residues contributed by p19 and the Alphabody, respectively. Thus, Alphabodies are suitable for targeting protein–protein interfaces of therapeutic importance and can be tailored to interrogate desired design and binding-mode principles via efficient selection and affinity-maturation strategies.

## Abstract (web summary)

Protein scaffolds can serve as alternatives to antibodies in a range of applications. Here, the authors report the design and development of Alphabody™, a protein scaffold featuring a single-chain antiparallel triple-helix coiled-coil fold that the authors use to develop Alphabodies that can neutralize human IL-23 with high specificity and affinity.

## Graphical abstract

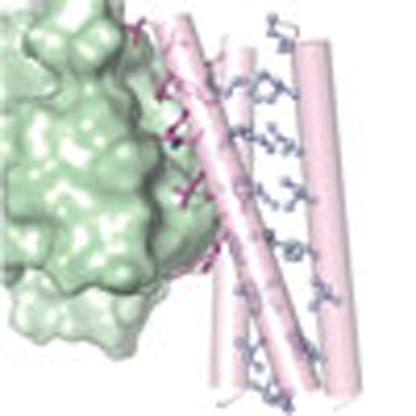

In the post-genomic era, the application and engineering of therapeutic antibodies to tackle cancer, as well as autoimmune and inflammatory disorders, has brought about a pronounced paradigm shift in the therapeutic targeting of protein–protein interactions^1^,^2^. At the same time, the elucidation of the molecular and structural basis of protein–protein interactions has emerged as the cornerstone for understanding the extra- and intra-cellular context of signalling pathways and for the rational design of molecules with antagonistic or agonistic behaviour against molecular targets of biomedical importance^3^. The inherent challenges associated with targeting protein–protein interfaces in a therapeutic setting^4^ have stimulated considerable efforts towards designed protein interactions^5^ and the development of engineered protein scaffolds that could serve as alternatives to antibodies in biomedical applications^6^,^7^. For instance, non-antibody molecular-binding platforms such as the DARPins^8^ Monobodies^9^, Anticalins^10^, Affibodies^11^, Affitins^12^ and the Adnectins^13^ have led to a large expansion of the structural repertoire of engineered protein scaffolds and have contributed significant added value in terms of their diverse physicochemical properties, pharmacokinetics and delivery to and through tissues of interest^6^.

The Alphabody scaffold is a computationally designed protein scaffold of about 10 kDa molecular weight, which was developed to serve as a therapeutic agent^14^. The scaffold does not have a counterpart in nature and is composed of a single contiguous polypeptide chain designed to adopt a triple-helix coiled-coil fold^14^. To explore the potential of the Alphabody platform in targeting biomedically relevant protein–protein interactions, we opted to target the pro-inflammatory cytokine interleukin (IL)-23, a well-established therapeutic target for the treatment of inflammatory diseases^15^. IL-23 is produced by dendritic cells and macrophages and is required for the survival and expansion of pro-inflammatory Th17 cells, which by virtue of their production of IL-17 are associated with the pathogenesis of autoimmune inflammatory disorders, such as multiple sclerosis, rheumatoid arthritis, psoriasis and inflammatory bowel disease^15^,^16^,^17^,^18^. In addition, IL-23 deficiency was recently shown to protect mice from tumour formation underscoring the general role of IL-23 in suppressing natural or cytokine-induced innate immunity and in promoting tumour development and metastasis^19^,^20^,^21^.

IL-23 adopts an atypical heterodimeric structure consisting of a p40 subunit encompassing three fibronectin-III-like domains, which is linked via a disulfide bond to an α-helical bundle subunit (p19) that topologically resembles long-chain helical cytokines^22^,^23^,^24^. IL-12, also a heterodimeric cytokine secreted by the dendritic cell to promote development of Th1 cells, also features the p40 subunit but the latter is coupled to a p35 subunit instead^15^. While both cytokines employ their p40 subunits to bind to IL-12Rβ1 as a common receptor, IL-23 uses its p19 subunit to engage its cognate IL-23R, whereas IL-12 binds to IL-12Rβ2 via the p35 subunit. Interestingly, the monoclonal antibody Ustekinumab, originally developed to neutralize IL-12 for the treatment of autoimmune inflammatory disorders, was subsequently shown to also antagonize IL-23 due to its ability to bind to the common p40 subunit employed by the two cytokines^25^,^26^,^27^,^28^,^29^. One of the reported side effects of the currently available anti-IL-12/IL-23 p40 therapeutic options is an increased susceptibility to infections, related to the important role IL-12 in mounting an appropriate immune system protection against pathogens^21^. In addition, several reports have described the protective role of and therapeutic potential of IL-12 in tumour development^20^,^30^,^31^.

We here report the design and development of Alphabodies as protein scaffolds not found in nature bearing unique physicochemical and structure–function properties, and probe their potential to serve as antagonists against pro-inflammatory human IL-23. We show that affinity-matured Alphabodies can bind with ultra-high affinity to IL-23, but not IL-12, via an extensive interaction interface that engages the p19 subunit of IL-23 to abrogate IL-23-mediated signalling *in vitro* and *in vivo*. Together, these findings establish the Alphabody as a potent and versatile protein-based scaffold and set the stage for their application in disrupting diverse protein–protein interactions of therapeutic relevance.

## Results

### Design and development of a reference Alphabody scaffold

Our endeavours towards the design of the Alphabody scaffold originated from the wealth of information on diverse types of coiled-coils^32^, as recorded in the CC+ database^33^. Initially, the Alphabody scaffold was launched as an assembly of three non-covalently associated peptides designed to form a parallel coiled-coil trimer^34^. This design was then redefined as a three-helix coiled-coil scaffold, wherein all three helices are contained within a single contiguous polypeptide chain. The rationale behind this effort has been manifold. First, the folding of a single-chain polypeptide, as opposed to peptidic associations, is not dependent on the concentration. Second, the designed scaffold needed to be producible by cost-effective and scalable recombinant protein production methods. Third, a single-chain construct allows independent definition of each amino-acid residue in any α-helix of the sequence. Fourth, a stable and autonomously folded protein can, in principle, allow accommodation of both conformational and linear binding epitopes.

**Figure 1:**
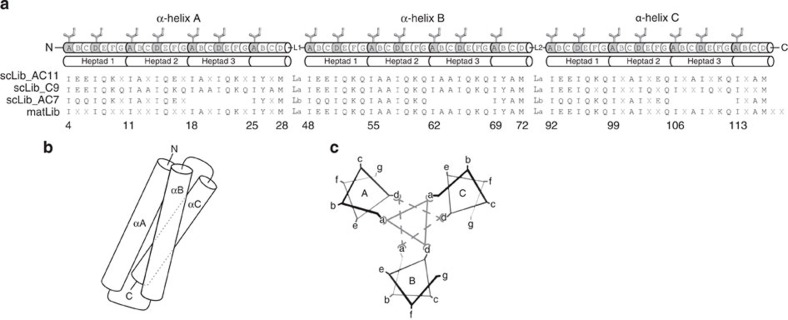
Schematic overview of the Alphabody platform. (**a**) Alphabodies are encoded by a single polypeptide featuring three α-helices (A–C) connected by linkers (L1, L2). The sequences of the three generic libraries (scLib_AC11, scLib_C9 and scLib_AC7) as well as the maturation library (matLib) used for biopanning are depicted. Amino-acid positions denoted with ‘x’ mark positions that were fully or partially randomized. The N terminus (N) of the Alphabody consists of the first 21 amino acids from the pelB leader sequence followed by an Asp in scLib_AC7 or Ser in all other cases. The linkers connecting the helices (L1 and L2) are identical within each library (La=T (GGSG)_4_MS, Lb=T(GGSG)_4_MD). The C terminus (C) differed between the generic libraries (TPGGSGGAAAHHHHHHGRAQ) and the maturation library (AAAHHHHHHQ) where in the latter the glutamine residue was encoded by an amber stop codon allowing for the translation of PIII fusion proteins. The sequence numbering corresponds to the maturation library, matLib. (**b**) Schematic rendering of the Alphabody scaffold as a single-chain three-helix antiparallel coiled-coil. (**c**) Schematic representation of the interactions between the core residues in an antiparallel coiled-coil. Hydrophobic interactions between isoleucines at positions a and d in each heptad repeat stabilize the Alphabody fold. Additional stabilizing interactions can arise from amino acids at positions e and g in helices A and C, positions g and g in helices B and C and positions e and e in helices A and B.

The reference scaffold, hereafter referred to as ‘scRef_L16’, was designed to feature three α-helices composed of four heptad repeats each, connected via glycine/serine-rich linkers (Fig. 1a). The reference sequence of each heptad repeat was defined as ‘I_a_A_b_A_c_I_d_Q_e_K_f_Q_g_’. Isoleucines were chosen at the heptad core positions (a and d) because of their specificity to induce three-stranded coiled-coil structures. Electrochemically neutral yet polar glutamines were placed at the interhelical ‘groove’ positions (e and g). Alanines were chosen at the exposed b- and c-positions because of their high α-helical propensity. Positively charged lysine was chosen at the most exposed f-position. Minor deviations from this heptad motif were introduced near the helical termini to improve capping. The full amino-acid sequence of scRef_L16 can be written as N-HRS1-L1-HRS2-L2-HRS3-C, wherein heptad repeat sequences HRS*x* have the sequence IEEIQKQIAAIQKQIAAIQKQIYRM and linker sequences L*x* are TGGSGGGSGGGSGGGSGMS (the capping residues ‘T’ and ‘MS’ at the N- and C termini are formally included in the linker segments).

Isoleucine core residues are known to have a high tendency to induce parallel coiled-coil trimers^35^. However, molecular modelling using the parallel Ile-stabilized trimer GCN4-pII (PDB code 1GCM)^35^ and the antiparallel Leu-stabilized coiled serine (PDB code 1COS)^36^ suggested that core residue layers with Ile at the a- and d-positions in two parallel helices could also be complemented by Ile core residues in a third, antiparallel helix (Fig. 1b,c). The linkers in scRef_L16 were therefore chosen to be long enough to allow antiparallel, as well as parallel folding (16 residues can theoretically bridge about 16 × 3 Å=48 Å, while 7 helical turns are 7 × 5.4 Å=38 Å in height). To explore the actual folding preferences in this regard, scRef_L16 and different variants were produced in soluble form and physicochemically characterized.

**Figure 2:**
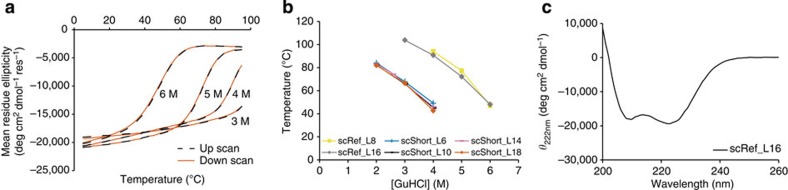
Stability and folding of the Alphabody scaffold. (**a**) Stability of the scRef_L16 reference Alphabody revealed by circular dichroism thermal denaturation experiments performed in 3–6 M guanidine hydrochloride (GuHCl). (**b**) Melting temperatures from thermal denaturation experiments as a function of GuHCl concentration. Alphabody scRef_L16 was tested together with scRef_L8, and compared with Alphabody scShort having four different linker lengths (L6–L18). (**c**) Circular dichroism wavelength scan on purified scRef_L16 confirming the correct folding and alpha-helical nature of the scaffold in its purified form.

His-tagged scRef_L16 and other variants described below were purified from *Escherichia coli* using metal-affinity and size-exclusion chromatography (SEC), with yields reaching several milligrams of pure protein per liter of culture. Circular dichroism measurements showed that scRef_L16 adopted a predominantly α-helical structure that was remarkably stable (*T*_m_ value of ~120 °C), as revealed by thermal denaturation studies in the presence of guanidine hydrochloride (Fig. 2a–c). Subsequently, a variant referred to as ‘scRef_L8’ was produced in which both linker segments were shortened by 8 amino-acid residues. Such linkers would be physically too short to connect the helical termini in a fully parallel coiled-coil structure. Surprisingly, we found that scRef_L8 and scRef_L16 were equally thermostable (Fig. 2b). This finding suggested that both constructs adopt an antiparallel configuration. However, it remained possible that an unfavourably short linker could induce local unwinding near the termini without causing an energetic cost in stability.

To examine the local unwinding hypothesis, a panel of variants was produced based on a scaffold variant carrying one fewer heptad repeat (referred to as ‘scShort’), equipped with linkers composed of 6–18 Gly/Ser residues (L6–L18). Consistent with the loss of two heptad core layers per construct, all scShort variants generally showed T_m_ values that were about 40 °C lower than the longer scRef constructs at comparable concentrations of GuHCl (Fig. 2b). The absence of any significant effect of the linker length in the context of a scaffold stabilized by only six core layers invalidated the local unwinding hypothesis and strengthened the notion of an antiparallel fold in an ‘up-down-up’ configuration.

### Design of Alphabody libraries

To explore the potential of scRef_L16 for generating target-specific binders, three random libraries were designed (Fig. 1a). A first library, denoted ‘scLib_AC11’, comprised six randomized c- and g-positions in helix A and five randomized b- and e-positions in helix C. These positions form a contiguous surface contributed by 11/14 (79%) of all b-, c-, e- and g-positions of the groove surface in between helices A and C (Fig. 1a–c). On the basis of the same design principle applied to the scShort sequence, the ‘scLib_AC7’ library was generated displaying four randomized positions in helix A and three in helix C. Both of these libraries are further referred to as ‘groove libraries’. A third library, ‘scLib_C9’, was derived from scRef_L16 and was intended to explore the surface functionality of helix C. Therefore, nine randomizations were introduced at b-, c- and f-positions in helix C. This library is further referred to as a ‘helix surface library’.

### Identification of Alphabody binders to human IL-23

The initial biopanning campaign employed the aforementioned generic libraries displayed as fusion constructs to the pIII protein of M13 phage. The library sizes ranged from 1.0 to 1.7 × 10^8^ unique clones. Enzyme-linked immunosorbent assay (ELISA) screening of output clones from consecutive biopanning rounds showed gradual enrichment of positive clones (optical densities (ODs) in screening ELISA at least three times higher than background), from about 33% in round 3 up to 95% in round 5 (Supplementary Fig. 1). Next, 91 positive clones from selection rounds 3, 4 and 5 were sequenced. We found that all binding clones originated from the groove libraries (scLib_AC11: 80 clones, scLib_AC7: 11 clones and scLib_B9: 0 clones). The population comprised 36 unique sequences, with some convergence (duplicates) already seen in round 3 and gradually increasing in the next rounds. Strikingly, the majority of sequences (56%) showed severe deletions primarily in helix B, with 5 clones (3 unique) lacking 2 heptad repeats, 35 clones (12 unique) lacking the entire helix B and 5 clones (3 unique) comprising only helix C. In addition, a marked enrichment in proline and amber stop codons was observed in the helix A, especially among clones lacking deletions in the sequence. The observed deficiencies in the sequences are likely due to the usage of the PelB signal sequence, which mediates post-translational translocation of protein to the bacterial periplasm through the Sec pathway. However, this signal sequence was previously shown to be inefficient for the display of fast-folding proteins^37^, as we have now also observed in the case of Alphabodies. Evidently, while defective sequences may be true binders as polypeptides with or without tertiary structure, their inherent instability renders them less-well controllable in terms of structural and physicochemical properties, and thus not well suited for further development. We have now resolved this issue in current work by substituting the PelB signal sequence with the DsbA signal sequence, which directs protein translocation to the periplasm via the co-translational signal recognition particle (SRP) pathway^38^,^39^.

Supplementary Table 1 shows the top-10 unique clones without B-helix deletions ranked according to binding performance in phage ELISA, the degree of competition by the neutralizing monoclonal antibody B-Z23 directed against the p19 subunit of IL-23 and the amino-acid residues observed at the variable positions. Apart from the Pro and stop codon enrichments, clone 72 had three helix-destabilizing glycines in helix A. In contrast, four clones showing nearly complete inhibition of binding to IL-23 in the presence of the neutralizing antibody B-Z23 (clones 52, 44, 59 and 33) were less affected by destabilizing elements (only clone 44 had a Pro at position 1 g). Further sequence comparisons elucidated the following apparent consensus binding motif: position A2g: aliphatic (M/I/V); A3c: small (A/G/S); A3g: aromatic (W/Y); C3b: aliphatic (V/I/L); C3e: aromatic (F/Y).

### Development of affinity-matured Alphabodies against IL-23

Clone 59 (hereafter referred to as ‘Cl59’) was purified as a soluble Alphabody following expression in *E. coli* and was subsequently tested in various binding and functional assays. We found that Cl59 bound with high affinity to human IL-23 (*K*_D_=3.8 nM in equilibrium ELISA and *K*_D_=7.0 nM in a kinetic ELISA assay). As described below, it also displayed moderate antagonistic activity in a functional assay on mouse splenocytes (*K*_I_=69 nM) (Table 1). In light of its promising initial characteristics, Cl59 was selected as the starting point for affinity maturation.

A maturation library termed ‘matLib’ (Fig. 1a; Supplementary Table 2) was established based on the sequence of Cl59 and the apparent consensus binding motif (Supplementary Table 1). This library was used to conduct two affinity-maturation campaigns in parallel. This resulted in a large panel of variants with improved apparent affinities in phage format, from which 14 unique clones were selected for further characterization in soluble format. To this set we added two consensus sequences from each campaign (‘MAcons’ and ‘MBcons’), three computationally designed variants (‘Cl59m’, ‘59m_C2eQ’ and ‘59m_A3cA_A4cS_C2eQ’) and the reference variant Cl59. Supplementary Table 3 lists the names and constitution of these 20 Alphabody variants in terms of selected residues at the variable positions, linker lengths and B-helix modification. All constructs were purified from *E. coli* with yields ranging from 0.37 to 10.1 mg per liter culture (mean=2.7 mg l^−1^).

**Figure 3:**
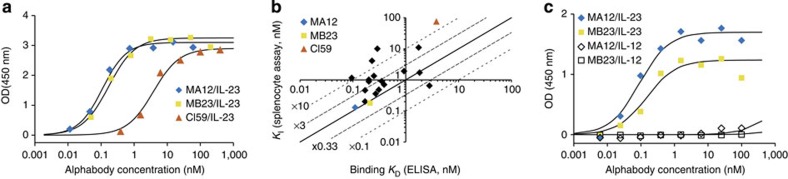
Binding properties of affinity-matured Alphabodies against human IL-23. (**a**) Comparison of ELISA binding profiles for matured Alphabodies from each maturation campaign against non-matured Cl59 Alphabody. (**b**) Comparison of *K*_I_ values obtained via the splenocyte functional assay and *K*_D_ values determined by equilibrium ELISA. Cl59, MA12 and MB23 are indicated. The thick diagonal straight line corresponds to *K*_I_=*K*_D_, thin straight lines correspond to *K*_I_ deviating by a factor 3 from binding *K*_D_. Dashed lines represent deviations by a factor 10. (**c**) Specificity of MA12 and MB23 towards human IL-23 versus human IL-12 determined by ELISA binding assays.

We employed a broad set of binding assays to characterize this set of 20 Alphabodies, which revealed that the vast majority of maturation clones (# 1–14) displayed subnanomolar affinities to IL-23 (Table 1). Both maturation campaigns yielded high-affinity binders (Fig. 3a). The highest affinity in standard ELISA assays was measured for MA12 and MA5 (*K*_D_=0.1 nM), which is a factor 38 better than the non-matured variant Cl59. Kinetic ELISA assays (Supplementary Fig. 2) and standard ELISA data (Fig. 3a) were globally in good agreement, as inferred from the consistency between sub- and supra-nanomolar affinities (Table 1). The main improvement in affinities appears to have arisen from an increase in the *k*_on_ by about one order of magnitude, with some *k*_on_ values exceeding 10^6^ M^−1^ s^−1^ (Table 1). Furthermore, *K*_D_ values measured via phage-coated ELISA were generally in good agreement with *K*_D_ values obtained for the soluble constructs, indicating that affinities determined in the early-screening phase of phage display are good predictors of the affinities of the soluble constructs.

### Antagonistic potency of affinity-matured Alphabodies

To assess the antagonistic capacity of the selected Alphabodies *in vitro*, a mouse splenocyte assay was established^40^. Functional inhibition constants (*K*_I_) were determined by measuring the inhibition of IL-23-induced IL-17 production in murine splenocytes. We observed that the majority of subnanomolar affinity binders also inhibited IL-23 with subnanomolar functional inhibition constants (Table 1). Over the whole range, the majority of *K*_I_ values deviated by less than a factor 3 from the binding *K*_D_ (median *K*_I_/*K*_D_=2.7) (Fig. 3b). Alphabody Cl59, which had the lowest affinity, also showed the weakest inhibition. The second best binder, MA12, showed the strongest inhibition capacity (*K*_I_=0.13 nM). This suggests that the functional inhibition potencies are predominantly determined by the binding affinities. In addition, we sought to characterize the specificity of binding of matured Alphabodies against human IL-23 compared with IL-12 by ELISA, as this could have therapeutic implications in targeting the functional dichotomy between these two pro-inflammatory cytokines. We found that both representative Alphabodies tested, MA12 and MB23, exhibited exquisite specificity towards IL-23 (Fig. 3c) suggesting that matured Alphabodies only target unique structural features in IL-23.

To confirm the binding and inhibition data in another cellular assay, the top-performing Alphabodies were tested on their capacity to neutralize IL-23-mediated signalling in a human DB lymphoma cell line^41^. For all Alphabodies tested, a clear dose-dependent inhibition of STAT3 phosphorylation was observed. Functional inhibition constants were determined using a calibration curve, in a similar manner as for the splenocyte assay. Here, Alphabody MB23 performed best, with a *K*_I_ of 0.16 nM, while most other variants were also found to have subnanomolar inhibition constants (Table 1).

**Figure 4:**
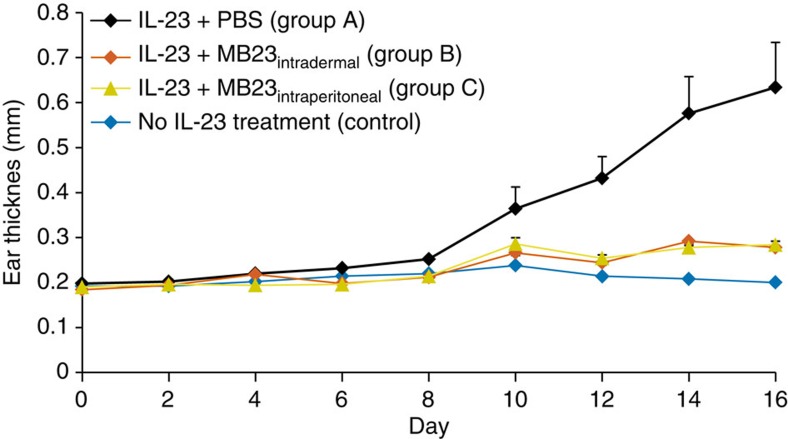
An affinity-matured Alphabody is able to prevent topical inflammation driven by IL-23 in mice. The ear thickness of 20 mice, subdivided into four groups (*n*=5), was measured as a function of time. Control, control group injected with PBS on days 1 to 15 every other day; Groups A–C, mice injected with human IL-23 according to the same scheme; groups B and C, mice that received injections with MB23-PEG on days −1, 0, 3, 6, 9, 12 and 15. Group B mice received intradermal injections of 10 μg of MB23-PEG and group C received intraperitoneal injections of MB23-PEG at 40 mg kg^−1^. Error bars represent the s.e.m.

In addition, we attempted to investigate the *in vivo* efficacy of one of the best binders, MB23, in a mouse model for skin inflammation induced by intradermal injection of human IL-23 into the ear^42^,^43^. In this model, repeat dosing with human IL-23 resulted in psoriasis-like thickening of the ear skin epidermis and swelling of the ear, which could be monitored *in vivo* by calliper measurements (Fig. 4). The negative-control group, which was injected with phosphate-buffered saline (PBS) showed no thickening, whereas the positive-control group injected with IL-23 only (group A) showed gradual thickening as of day 8. Mice in groups B and C were injected with both IL-23 and MB23, which was PEGylated (40 kDa linear polyethylene glycol (PEG)) via a cysteine residue introduced in the B-helix of the Alphabody. Group B received intradermal MB23 injections and Group C was injected intraperitoneally. Indeed, both MB23-treated groups showed the same ear-thickness curves, only slightly above the negative-control curve, indicating that MB23 treatment effectively suppressed IL-23-induced ear swelling (Fig. 4).

### Structure of Alphabody MA12 and its complex with IL-23

**Figure 5:**
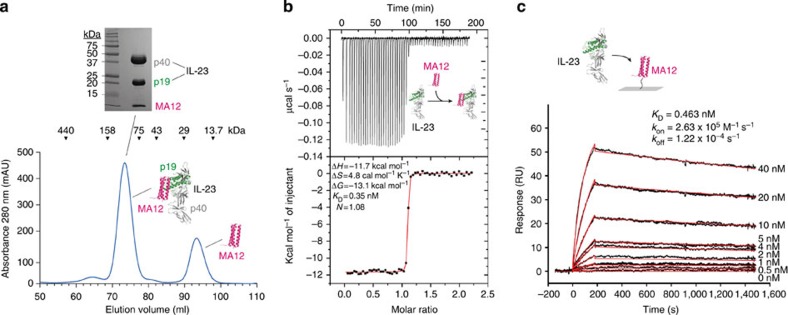
Biochemical and biophysical characterization of the IL-23:MA12 interaction. (**a**) Size-exclusion chromatography profile for the isolation of IL-23:MA12 complex in the presence of an excess of MA12 Alphabody. Elution volumes of protein standards are indicated at the top. The inset shows a Coomassie-stained SDS–polyacrylamide gel electrophoresis gel corresponding to the IL-23:MA12 complex elution peak. Molecular weights of protein standards are indicated. (**b**) ITC thermogram and analysis of the titration of IL-23 (4.9 μM in the microcalorimeter cell) with the MA12 Alphabody (53.2 μM in the titration syringe). Data were fitted to a ‘single-site binding model’, giving the apparent molar reaction enthalpy (Δ*H*°), entropy (Δ*S*°), Gibbs free energy (Δ*G*°), dissociation constant (*K*_D_) and stoichiometry of binding (*N*) of complex formation. (**c**) SPR sensorgrams for the association of recombinant human IL-23 (0.5–40 nM) with immobilized MA12 Alphabody (43 RU) were fitted to a 1:1 Langmuir binding model, giving the apparent dissociation constant (*K*_D_), association rate constant (*k*_on_) and dissociation rate constant (*k*_off_) of complex formation.

To obtain structural insights into the three-dimensional structure of the Alphabody scaffold and the structural and binding principles underlying its neutralizing interaction with IL-23, we carried out crystallographic studies of human IL-23 in complex with MA12 complemented by isothermal titration calorimetry (ITC) and surface plasmon resonance (SPR). Human IL-23 was transiently expressed by co-transfecting IL-12 subunit beta (p40) and IL-23 subunit alpha (p19) in HEK239T cells in the presence of kifunensine to limit the extent of N-linked glycosylation^44^. N-linked glycosylation on IL-23 was further trimmed during purification by treating IL-23 with EndoH. The free cysteine of the MA12 Alphabody was blocked by iodoacetamide and the N-terminal His-tag was removed by trypsin. Mixing of purified human IL-23 with an excess of purified MA12, followed by SEC, yielded a highly monodisperse fraction containing the IL-23:MA12 complex (Fig. 5a). ITC experiments showed that MA12 binds to IL-23 with subnanomolar affinity (*K*_D_=0.35 nM) ), in what emerged as an enthalpically driven interaction with a 1:1 stoichiometry (Fig. 5b). Such binding affinity is consistent with the ELISA-based affinity determinations (Table 1). In addition, kinetic binding parameters obtained by SPR indicated a relatively fast on-rate (*k*_on_=2.6 × 10^5^ M^−1^ s^−1^) and a slow off-rate (*k*_off_=1.2 × 10^−4^ s^−1^), yielding a *K*_D_=0.46 nM, in good agreement with values obtained from all other binding assays employed (Fig. 5c; Table 1).

**Figure 6:**
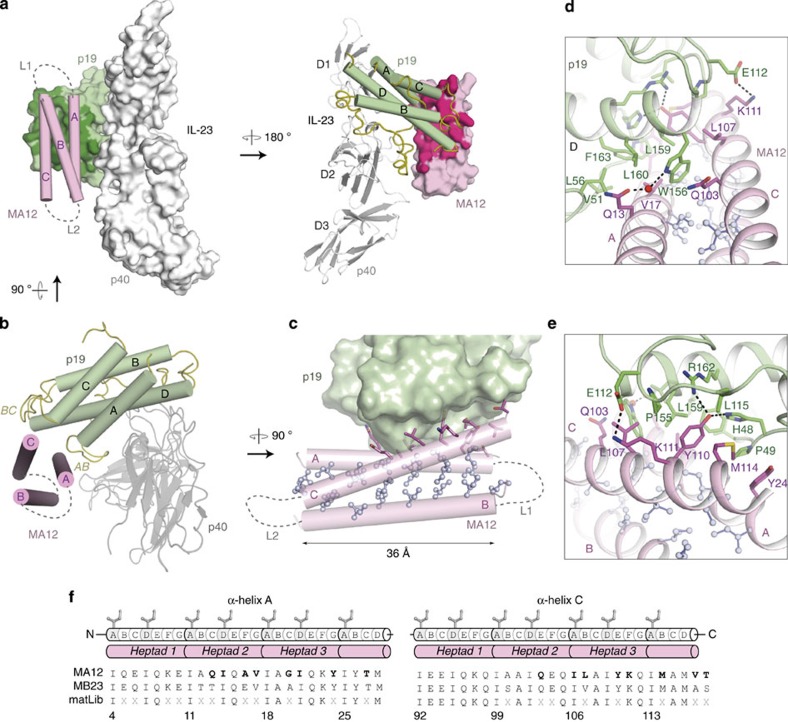
Structural analysis of the IL-23:MA12 complex. (**a**) Assembly of the IL-23–MA12 complex. (Left) the IL-23:MA12 complex with IL-23 in surface mode and the MA12 Alphabody in cartoon mode. (right) The complex is rotated by 180° and IL-23 is shown in cartoon mode and MA12 in surface mode. MA12 is coloured in pink, p19 in green and p40 in grey. The MA12-interacting surface of p19 (800 Å^2^) is indicated in dark green and the p19-interacting surface of MA12 (840 Å^2^) in magenta. The linker regions between helices A and B (L1) and B and C (L2) in MA12 are represented as dashed lines. (**b**) Top view of the IL-23–MA12 complex. MA12 interacts with helix D and the AB and BC loops of p19. (**c**) View of the isoleucine core of the Alphabody core in MA12 and engagement of the p19 subunit via the interhelical groove presented by helices A and C of MA12. (**d**) Detailed view of the IL-23:MA12 interactions around residue W156 in the p19 subunit of IL-23. (**e**) Detailed view of the interactions of MA12 helix C with p19. Residue numbering in the structure of human IL-23 reported herein reflects the sequence numbering of the protein in Uniprot. Thus, residue numbers in the p19 subunit of human IL-23 differ by 19 with respect to equivalent residues in PDB entries 3DUH, 3D85, 3D87, 3QWR and 4GRW (for example, W156 in the p19 subunit of human IL-23 is equivalent to W137 in previously reported structures). (**f**) Comparisons of sequences corresponding to helices A and C in the two best matured Alphabodies MA12 and MB23, against the reference sequences in matLib. Positions labelled with ‘x’ corresponding to variable amino-acid position. Amino acids in MA12 that are involved in binding to human IL-23 are shown in bold.

Crystallization trials of highly monodisperse preparations of the IL-23:MA12 complex yielded two distinct crystal forms that allowed us to determine crystal structures at 1.74 and 3.4 Å resolution (Table 2), providing the opportunity to visualize the binding epitope in detail and to cross-validate structural observations. MA12 folds into a left-handed triple-helix coiled-coil structure, with the binding surface located mainly at the C-terminal half of helices A and C (Fig. 6a,b). The same structure and binding mode to IL-23 are maintained in both crystal forms (Supplementary Fig. 3). In agreement with the ITC-derived stoichiometry of the complex, one copy of MA12 binds to the p19 subunit of IL-23 and is oriented parallel to the longitudinal axis of the p40 subunit (Fig. 6a,b). The respective binding interfaces feature 20 amino-acid residues contributed by MA12 and 28 amino-acid residues contributed by p19, burying a total surface area of 800 and 840 Å^2^, respectively (Fig. 6a; Supplementary Table 4). This MA12 interface area constitutes 14.4% of the total surface area of the Alphabody. Five hydrogen bonds and two salt bridges are formed upon binding, while the rest of the interaction interface can be described in terms of van der Waals contacts (Supplementary Table 4).

The binding interface is further hallmarked at its two ends by two prominent attachment points. At one end, Trp156 from IL-23 p19, a residue that was previously proposed to define a hotspot mediating the cognate interaction of IL-23 with IL-23R (ref. 24), pokes the benzene ring part of its indole ring into the groove defined by helices A and C of the Alphabody, to interact with the Ile106 core residue (Fig. 6d). At the same time, the N–H group of the indole moiety engages in a water-mediated hydrogen bond with Gln13 projecting from helix A of MA12 (position A2c) (Fig. 6d). At the other end, Tyr110 on MA12, a conserved residue in all matured binders (Supplementary Table 3), inserts into a pocket on IL-23 p19 defined by the BC and AB loops and the N-terminal part of helix D (Fig. 6e). Furthermore, we note that this segment of the AB loop in the p19 subunit of IL-23 is disordered in all currently available crystal structures of IL-23 (refs 22, 23, 24, 45), suggesting that the interaction with the Alphabody induces the observed conformation. Contrary to helices A and C of MA12, helix B does not participate at all in the binding epitope with p19 (Fig. 6). The glycine-rich linkers connecting the three helices could not be visualized in the electron density maps and were not included in the model. Importantly, comparison of the variable positions in MA12 involved in the binding interface with IL-23 with those of MB23, another well-performing Alphabody, reveals strong sequence conservation levels (Fig. 6f and Supplementary Table 3). Thus, such strong sequence conservation patterns in residues involved in contacts with IL-23 and the very similar binding profiles shared by the best binders (Table 1), essentially warrant that all matured high-affinity binders bind using similar structural principles.

We were intrigued by persisting residual difference electron density emanating from the Cδ1 position of Trp319 in IL-23 p40 up to the last rounds of refinement. Given previous reports that IL-12, a cytokine related to IL-23, can be C-mannosylated via a tryptophan residue^46^, we wondered whether this would also be the case for IL-23. Indeed, mass spectrometry on tryptic peptides of our recombinant human IL-23 confirmed that Trp319 in the p40 subunit of IL-23 is mannosylated (Supplementary Fig. 4a,b). However, modelling of a C-linked mannose to Trp319 proved challenging due to the likely structural heterogeneity of this adduct^47^, prompting us to omit the sugar moiety from the final models.

Finally, we note that the engineered Cys60, located in the middle of helix B of MA12 and all other affinity-matured Alphabodies (Supplementary Table 3), remains freely accessible to solvent well away from the interaction epitope and is well positioned for introducing potential post-translational chemical modifications to improve bioavailability and modulate physicochemical properties. For example, the 40-kDa PEG-moiety attached to the MB23 Alphabody in the *in vivo* study on mice can be expected not to interfere with the binding, as also evidenced by *in vivo* data.

## Discussion

The growing need for therapeutics that can efficiently antagonize biomedically relevant protein–protein interactions has stimulated the expansion of the repertoire of novel and repurposed protein scaffolds. Despite good progress in the development of antibody-based^48^ and non-antibody scaffolds^6^ to serve as therapeutic biologicals in the areas of cancer, autoimmune diseases and inflammatory disorders^1^, the available approaches are yet to reach their full potential, as many drug discovery/development programmes are faced with high attrition rates^49^.

Here we reported on the development of a novel protein scaffold, termed the Alphabody, which emerged from a combination of *de novo* design and experimental validation. Alphabody sequences were found to fold as antiparallel triple-stranded α-helical coiled-coil structures, thus adopting a previously unknown fold. The core of the antiparallel coiled-coil fold features tightly packed isoleucines at conventional heptad a- and d-positions with a regular ‘knobs-into-holes’ configuration, wherein the a- and d-residues of the antiparallel B-helix form core layers together with d- and a-residues, respectively, from the two parallel helices A and C. This type of packing is similar in configuration, yet distinct at the atomic level, from the leucine-stabilized antiparallel three-stranded ‘coiled serine’ structure^36^, and possesses exceptional thermostability. Thus, such near-optimal core packing and the repetitive nature of the constituting heptad repeats allow for a scalable scaffold that is insensitive to multiple substitutions, thereby enabling the design of generic libraries for phage display that can exploit the entire scaffold surface, rather than the loop regions, as is the case for most other scaffold proteins. More specifically, Alphabodies can carry up to 25 variable positions that can be optimized for binding and other properties. This offers important advantages in the therapeutic targeting of protein–protein interfaces, an area of increasing significance in drug development. In addition, the possibility to combine several tailor-made properties, including optimization of loop regions, on such a robust scaffold offers opportunities to develop Alphabodies that are minimally immunogenic, paving the way for (pre)clinical development of the scaffold. Current approaches to predict and assess the immunogenicity of protein therapeutics range from computational strategies to screening against human leukocyte antigen class II molecules, cellular models employing CD4+ T cells, as well as transgenic animal models including immunodeficient mice reconstituted with human hematopoietic stem cells^7^,^50^,^51^.

Our Alphabody libraries were subdivided into structural ‘themes’, focusing on either the concave groove surface formed by the A- and C-helices, or the convex B-helix surface. The biopanning campaign against the present target, IL-23, using a mixture of the libraries converged towards groove binders only. Importantly, matured Alphabody variants were shown to potently suppress IL-23-mediated inflammatory phenotypes *in vitro*, with the best-performing Alphabodies reaching subnanomolar affinities in various binding assays (Table 1; Fig. 4). The observed kinetic binding profiles of both the initial clones and the matured Alphabodies are within ranges observed for high-affinity protein–protein interactions (*k*_on_~10^5^–10^6^ M^−1^ s^−1^ and *k*_off_ values ~10^−4^–10^−5^ s^−1^) (Table 1). In this regard, it was surprising to see that the gain in binding affinity in matured Alphabodies appeared to result primarily from improvements in the association rate constants by about an order of magnitude (Table 1). In combination with their favourable physicochemical properties, such binding potencies promise to render Alphabodies as multi-purpose anti-inflammatory agents for the treatment of inflammatory disorders such as rheumatoid arthritis, inflammatory bowel disease and multiple sclerosis^15^. This was partly validated in this study by the successful *in vivo* results obtained via intradermal and intraperitoneal injection of MB23-PEG in mice. Furthermore, the fact that the developed Alphabodies exclusively target the p19 subunit of IL-23 and show no cross-reactivity with IL-12, offers the opportunity to decouple therapeutic modulation of signalling pathways mediated by IL-23 from those mediated by IL-12 (ref. 15).

**Figure 7:**
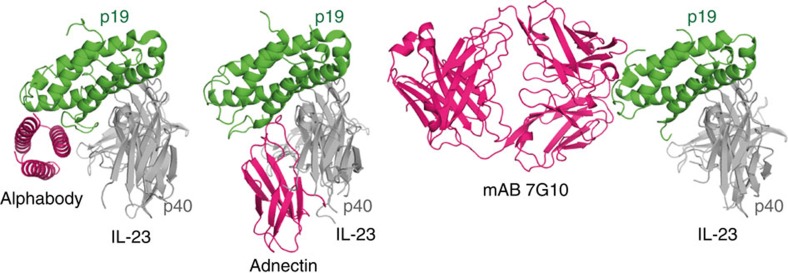
Comparison of binding modes of human IL-23 with antagonists. Comparison of structures for different IL-23:antagonist complexes: Alphabody:IL-23 complex, adnectin:IL-23 complex (pdb code 3QWR)^45^ and 7G10 antibody:IL-23 complex (PDB code 3D85)^22^. The complexes are oriented based on structural superpositions against the p19 subunit of human IL-23.

The design principles employed to develop the Alphabody scaffold and subsequent libraries were borne by our structural data. Arguably one hallmark of the Alphabody platform is the way it employs the surface defined by two of the scaffold helices to target a hitherto unexploited binding site on the p19 subunit of human IL-23 (Figs 6 and 7). The binding mode observed in the complex of MA12 with human IL-23 is strongly reminiscent of the principles employed in the two-sided design of a *de novo* binding pair^52^. In this binding mode, aromatic side chains with amphipathic character, such as tryptophan and tyrosine, are mutually contributed by the binding pair to mediate both aromatic and H-bonded interactions. Interestingly, Alphabody MA12 sequesters Trp156 on human IL-23 (Fig. 6), a residue that has been proposed as a hotspot for the binding of cognate IL-23R (ref. 24).

The potential to exploit the Alphabody scaffold to target diverse binding epitopes via classical biopanning and selection approaches and/or computational design is intrinsically large. Computational approaches are steadily emerging but are still lacking the robustness of experimental strategies based on screening and selection. The α-helical nature of the Alphabody scaffold, however, makes it particularly amenable to design techniques such as the grafting of binding motifs from natural α-helical ligands, at least in cases for which structural templates are available. The present study also illustrates how initial drug discovery can be accomplished by exploring different thematic libraries. Subsequently, once a preferred type of binding surface has been determined (for example, a groove, helix or a loop) and initial binding sequences are available, the rigid α-helical nature of the scaffold will essentially allow modelling of individual sequences onto a scaffold template structure with reasonable accuracy. In addition, the rigidity of the Alphabody helices may provide an entropic advantage when the helical surfaces are structurally compatible with the binding epitope. We envisage that such design options will considerably facilitate the rational design of focused libraries for further affinity maturation. Finally, having delineated a given critical binding region, the remainder of the scaffold can be independently optimized for other purposes such as solubility enhancement, biospecificity, labelling and PEGylation.

## Methods

### Recombinant production of Alphabodies

The complementary DNA (cDNA) of Alphabodies was cloned as N-terminally tagged decahistidine fusion proteins into the pET16b-vector (Novagen). Recombinant production was performed in transformed BL21(DE3)pLysS cells (Life Technologies), grown in Luria-Bertani (LB) medium at 37 °C. Expression was induced by adding 1 mM isopropylthio-β-galactoside at an OD_600 nm_ of 0.6 and expression was continued for 4 h at 37 °C. Cells were harvested by centrifugation and resuspended in 50 mM Tris, 500 mM NaCl pH 7.8 and frozen at −80 °C. Cell lysis was performed by thawing and sonication. 4-(2-aminoethyl) benzenesulfonyl fluoride hydrochloride (0.1 mM) was added to the lysed cells, together with 10 μg ml^−1^ DNAseI and 5 mM MgCl_2_. The suspension was centrifuged for 20 min at 40,000 *g*. Typically, Alphabodies were purified from the soluble fraction, which was loaded on a 5-ml IMAC HP Ni sepharose column (GE Healthcare) and eluted with an imidazole gradient over 15 column volumes. Final polishing and desalting was performed on a Superdex 75 size-exclusion column (GEHealthcare) equilibrated in PBS.

### Development of Alphabody libraries

Genes with position-specific randomization (NNK) were designed wherein the Alphabody sequences were fused N-terminally to the pIII protein of bacteriophage M13. These libraries were ordered from GeneArt AG (Germany) and delivered as transformed *E. coli* cells (strain ER2738, supE strain). The library sizes ranged from 1.0 to 1.7 × 10^8^ unique clones. Alphabody displayed on pIII of the phagemid was confirmed by western blot analysis using anti-pIII (MoBiTec GmbH) and amounted to about 10% of total pIII protein.

### Biopanning procedure

A biopanning campaign was set up using a mixture of equal amounts of the three libraries scLib_AC11, scLib_AC7 and scLib C9. A soluble panning format was applied, that is, phages were pre-incubated with the target and subsequently captured on streptavidin beads. The preincubation was performed during 1 h at room temperature (RT) on an end over end shaker, with hIL-23 (Biolegend) bound to biotinylated anti-IL-23p40 antibody (Biolegend) in a 2:1 molar ratio in PBS supplemented with 2% skimmed milk. The capturing step was performed using Dynabeads (50 μl). Beads were washed 10 times with 1 ml of 0.1% Tween 20 in PBS. Elution was performed with 200 μl of buffer (100 mM glycine–HCl, pH 2), and the eluate was neutralized by adding 50 μl of 1 M Tris pH 8.0. Five consecutive panning rounds were conducted wherein the hIL-23 target concentration was decreased as of round 3 (rounds 1 and 2: 200 nM, rounds 3–5: 100, 10 and 1 nM, respectively). Phagemid was propagated as follows. Neutralized phagemid solution was added to 1.75 ml of LB. After taking out 50 μl for further titration, the rest of the phagemid solution was added to 18 ml of TG1 cells that had already grown to the exponential phase (OD_600 nm_=0.5). After 30 min incubation at 37 °C, the solution was centrifuged and the pellet was resuspended in 250 ml of LB medium+ampicillin+glucose. Overnight (O/N) incubation was performed at 37 °C (180 r.p.m.).

### Screening and competition ELISA

Phage ELISA was used to screen for positive clones and to check which of them showed competition with the neutralizing antibody B-Z23, as follows. Nunc ELISA plates were coated with 100 μl per well neutravidin (10 μg ml^−1^) in PBS for 1 h at RT. In the following steps, the plate was washed 5 times with PBS, 0.05% Tween 20, between each incubation step. The plate was blocked O/N at 4 °C with 200 μl per well of PBS, 0.5% gelatin and 0.1% BSA. Biotinylated anti-human-p40 (100 μl, 100 nM, Biolegend) in 0.1% BSA–PBS was added for 1 h at RT. For the competition ELISA, 150 nM hIL-23 (Biolegend) was pre-incubated with 150 nM antibody B-Z23 (CellSciences) in 0.1% BSA–PBS for 1 h at RT. For the screening ELISA, the same sample without B-Z23 antibody was used. Then, 66.6 μl of this sample (final concentration 100 nM) and 33.3 μl of rescued phagemid supernatant was added to the plate for 2 h at RT. Bound phagemid was detected with anti-M13-HRP (GE Healthcare) for 0.5 h at RT followed by 3,3′,5,5′-tetramethylbenzidine (TMB) (Sigma) staining. The colour reaction was stopped with 100 μl 0.5 M H_2_SO_4_.

### Phagemid rescue

TG1 cells, infected with selected phagemid, were titrated on an agar plate (2 × TY, agar, ampicillin). Individual clones were picked from the agar plate and cultured O/N in a microtiter plate containing 100 μl 2 × TY AG per well^53^. One μl of each O/N culture was transferred to a microtiter plate containing 100 μl of 2 × TY, 0.1% glucose, 100 μg ml^−1^ ampicillin and 10^8^ helper phage per well and grown at 37 °C for 2 h while shaking. Kanamycin/ampicillin (30 μl) was added to each well to a final concentration of 100 and 30 μg ml^−1^, respectively. After O/N incubation at 30 °C while shaking, plates were spun (611*g* for 20 min), and supernatant containing phagemid expressing Alphabody was used directly in ELISA.

### ELISA assays

*Phage-coated ELISA*. To screen for IL-23-positive clones, we developed a phage-coated ELISA method. First, 100 μl anti-M13 at 5 μg ml^−1^ in PBS was coated O/N at 4 °C (VWR, 27942001) onto a Nunc Maxisorp microtiter plate (Thermo Scientific, 42404). Then the plate was blocked with 130 μl PBS, 0.1% BSA, 0.5% gelatin for 1 h at RT. Between incubations, plates were washed with PBS containing 0.05% Tween 20. After addition of 70 μl PBS with 0.1% BSA to the washed wells, 30 μl phagemid from a 96-well rescue of clonal phagemid was added. Following an incubation of 1 h at RT and a washing step, 1 nM IL-23 (eBioscience, 34-8239-85) was added for 1 h at RT. Then after washing, 100 μl biotinylated anti-p40 in PBS 0.1% BSA (Imtec Diagnostics, BE 505302) was added. For each clone, the background (without IL-23) was measured as well. After washing, staining was performed with 100 μl streptavidin-HRP in PBS 0.1% BSA (Biolegend, 405210) followed by a TMB reaction that was stopped with 0.5 M H_2_SO_4_. The OD values were measured at 450 nm (OD_450_). A clone was scored as positive when OD_target_>3 × OD_background_.

To measure the affinities of the Alphabodies at the phagemid level, we optimized the phage-coated ELISA as described above and incubated different dilutions of target O/N at RT with the coated phagemid. Phagemid (30 μl in 70 μl PBS, 0.1% BSA) was coated indirectly via anti-M13 as described above, and IL-23 was diluted in a concentration range from 10 to 0.0098, nM (1/5 dilutions) in PBS, 0.1% BSA, and 100 μl was added to the plate after washing away the non-bound phagemid. In the phage-coated ELISA format, the phagemid with IL-23 incubation was carried out O/N at RT. IL-23 binding was detected with biotinylated anti-p40 and streptavidin-HRP.

*Standard and kinetic ELISA assays*. Neutravidin (10 μg ml^−1^ in PBS) was coated onto a Nunc plate for 1 h at RT. Plates were washed 5 × with PBS+0.05% Tween 20 and blocked O/N with 0.1% BSA+0.5% gelatin at 4 °C. Biotinylated anti-p40 antibody (Imtec Diagnostics, 10 nM in PBS+0.1% BSA) was incubated for 1 h at RT, followed by washing. Next, human IL-23 (eBioscience, 10 nM in PBS+0.1% BSA) was incubated for 1 h at RT while shaking, followed by washing. For the standard ELISA, fivefold dilution series of Alphabodies in PBS+0.1% BSA were incubated for 24 h while shaking (plates meanwhile covered with parafilm), followed by washing. For the kinetic ELISA, the same dilution series was added at different time points (24, 3, 1 h, 15, 8 and 4 min) before the washing step; wells not yet filled with Alphabody were temporarily filled with PBS+0.1% BSA. Both types of ELISA were developed with anti-penta-His-HRP (1/1,000 in PBS+0.1% BSA) for 1 h at RT, while shaking. Further steps were as described for the phage-coated ELISA.

Kinetic binding data in the context of kinetic ELISA assays were derived as follows: ODs were divided by the maximum OD values to obtain the fraction of bound IL-23 (vertical axis in Supplementary Fig. 3). Each full data set was fitted with only two parameters, *K*_D_ and *k*_on_, to yield *k*_off_.

### Mouse splenocyte assay

To assess the inhibitory capacity of the selected Alphabodies in an *in vitro* assay, a mouse splenocyte assay was set up^40^. Human IL-23 in the presence of IL-2 stimulates the production of IL-17 in murine splenocytes at very low (picomolar) concentrations, which can be inhibited by co-incubation of inhibitors against either p40 or p19. The IL-17 response (measured using a Quantikine ELISA kit, R&D Systems) was determined in parallel for a calibration experiment with increasing hIL-23 concentrations and for an inhibition experiment using a fixed (2.6 pM) IL-23 concentration and increasing Alphabody concentrations (fivefold dilutions in the range 0–1 μM). The inhibition constants *K*_I_ were determined by fitting the data to a nonlinear equation describing the relationship between OD values and total Alphabody concentrations. The calibration and inhibition experiments were performed in fourfold and eightfold, respectively, on the same ELISA plate (data were averaged).

### DB cell assay

hIL-23 induces STAT3 phosphorylation upon engagement with IL-23R at the surface of DB (human lymphoma) cells (DSMZ, Germany). A calibration curve was established by determining the level of STAT3 phosphorylation in DB cells as a function of added human IL-23 within a concentration range of 0.01–10 nM. To measure the inhibitory activity of the hIL-23-binding Alphabodies, varying concentrations of Alphabodies (500–0.01 nM) were pre-incubated with a fixed concentration of hIL-23 (200 pM) and pre-incubated samples were added to DB cells for 1 h, in parallel to the calibration curve samples. Subsequently, DB cell lysates were prepared for STAT3 phosphorylation measurement by sandwich ELISA (PathScan ELISA). Inhibition curves were fitted to derive inhibition constants *K*_I_ of the tested Alphabodies.

### Psoriasis animal model

To determine the ability of affinity-matured Alphabodies to antagonize IL-23-driven inflammation *in vivo*, a 40-kDa linear PEGgylated form of variant MB23 (MB23-PEG) was tested in a mouse ‘ear model’^42^,^43^. Local ear skin inflammation was induced by repeated intradermal administrations of human IL-23 (1 μg) every other day within a period of 15 days into the right ear pinna of 20 male C57Bl/6J mice (Harlan Laboratories, Horst, Netherlands) that were 10 weeks old at the start of the experiment. The control group received intradermal PBS injections under the same administration scheme. MB23-PEG was administered by intradermal (10 μg; Group B) or intraperitoneal (40 mg kg^−1^; Group C) administrations on days −1, 0, 3, 6, 9, 12 and 15. Group A mice were not administered MB23-PEG, but PBS instead as a vehicle control. Skin swelling was measured by determining the ear skin thickness with a caliper on day 0 and subsequently every other day until day 16. The animal studies were approved by the Ethics Committee for Animal Protection under the jurisdiction of the regional administrative authority of Karlsruhe, Germany.

### Recombinant proteins for crystallographic studies

For large-scale production cDNA for the MA12_B2fC Alphabody was cloned into the pET16b-vector (Novagen) in frame with a N-terminal decahistidine tag. Recombinant production was performed in transformed BL21(DE3)pLysS cells, grown in LB medium at 37 °C. Expression was induced by adding 1 mM isopropylthio-β-galactoside at an OD_600 nm_ of 0.7 and expression was continued for 4 h at 37 °C. Washed inclusion bodies were solubilized in 6 M guanidine hydrochloride (GuHCl), loaded on a Ni Sepharose column, washed with buffer devoid of GuHCl, and eluted with imidazole. Next, the desalted protein sample was incubated for 2 h at 37 °C with magnetic trypsin beads to remove the N-terminal His-tag. The free cysteine of the MA12_B2fC Alphabody was blocked for 15 min by incubating with 5 mM iodoacetamide. Finally, as a polishing step the sample was injected on a Superdex 75 column equilibrated with 20 mM HEPES, 150 mM NaCl, pH 7.4.

Human IL-23 was produced by transient expression in mammalian cells^23^. cDNAs corresponding to the human IL-23 subunits, p19 (residues 1–189) and p40 subunits (residues 1–328) were purchased from GeneArt and cloned into the pHL expression vector^54^ between the EcoRI and KpnI sites. The p19 subunit was cloned in frame with the C-terminal His-tag, while the p40 expression construct carried a stop codon before the KpnI site. Both p19 and p40 cDNA sequences corresponded to the reference sequences in the NCBI databank, NM_016584.2 and NM_002187.2, respectively. However, the AAT codon for Asn248 in the p40 sequence was replaced with AAC to remove an EcoRI site. Large-scale expression experiments in human embryonic kidney 293T (HEK-293T) cells were conducted in roller bottles. HEK-293T cells grown in roller bottles were co-transfected with the pHL-p19-His and pHL-p40 expression constructs in a 1:1 ratio, using polyethyleneimine as transfection reagent. Expression was performed in serum-free Dulbecco's modified Eagle's medium/F12 medium supplemented with 5 μM kifunensine. Human IL-23 was purified from the conditioned medium by immobilized metal-ion affinity chromatography using Talon matrix (Clontech) followed by SEC using a Superdex 200 column (GE Healthcare).

### Crystal structure determination of the IL-23:MA12 complex

The IL-23:MA12 complex was formed by adding a molar excess of the MA12_B2fC Alphabody to recombinant human IL-23 that had been partially deglycosylated O/N with Endoglycosidase H (New England Biolabs). The complex was isolated from the excess of Alphabody by SEC using a Superdex 200 column with 20 mM HEPES, 150 mM NaCl, pH 7.4 as running buffer, and concentrated by ultracentrifugation to 6 mg ml^−1^. Nanolitre crystallization trials were set up at RT using a Mosquito crystallization robot (TTP Labtech) against commercially available sparse matrix screens (Hampton Research and Molecular Dimensions). Numerous hits were obtained in the PEG/ION HT screen. Additional hits were found in various conditions of the ProPlex HT and Crystal Screen HT screens.

Single crystals from optimized conditions were transferred to a drop of reservoir solution with the use of a nylon loop mounted on a SPINE standard cryocap, and cryoprotected with 20% PEG 400 before being flash-frozen in liquid nitrogen. Diffraction experiments were conducted on the X06SA and X06DA beamlines at the Swiss Light Source (Paul Scherrer Institute, Villigen, Switzerland). Data were integrated and scaled using the XDS suite^55^. Crystals belonging to crystal form 1 were grown from 20.75% (w/v) PEG 3,350, 200 mM Na_2_SO_4_. Crystals belonging to crystal form 2 were grown from 20.75% (w/v) PEG 3,350, 200 mM potassium formate. The structure of the IL-23:MA12_B2fC complex was determined by maximum-likelihood molecular replacement as implemented in the program suite PHASER^56^, using the structure of human IL-23 (PDB entry 3DUH)^24^ as a search model and structure-factors derived from X-ray diffraction data measured from crystal form 1 crystals. Model (re)building was carried out manually using the program COOT^57^. Crystallographic refinement and structure validation were carried out using PHENIX^58^.

Buried surface areas and analysis of interaction interfaces were calculated via the PISA server^59^. Rendering of the structures as illustrated in Figs 3, 4, 5 and Supplementary Figs 4 and 5a was carried out in PyMOL^60^.

### ITC

For calorimetric measurements, recombinant human IL-23 and the MA12_B2fC Alphabody were prepared by SEC in the same running buffer (20 mM Hepes, 150 mM NaCl, pH 7.4). Protein concentrations were measured spectrophotometrically at 280 nm using calculated theoretical extinction coefficients, and all solutions were extensively degassed before use. Experiments were carried out using a VP-ITC MicroCalorimeter (MicroCal, MA) at 30 °C, and data were analysed using the Origin ITC analysis software package supplied by MicroCal. Human IL-23 (4.9 μM in the microcalorimeter cell) was titrated with MA12 Alphabody (53.2 μM in the titration syringe). Titrations were preceded by an initial injection of 3 μl, and were carried out using 5-μl injections applied 300 s apart, under constant stirring. The thermal titration data were fitted to the ‘one binding site model’, and apparent molar reaction enthalpy (Δ*H*°), apparent entropy (Δ*S*°), dissociation constant (*K*_D_) and stoichiometry of binding (*N*) were determined.

### SPR

SPR experiments were carried out using a Biacore 3,000 instrument at 25 °C with HBS-EP pH 7.4 (GE Healthcare) as running buffer. Forty-three RU of MA12_B2fC were immobilized on a CM5 chip by amine coupling. Recombinant human IL-23 (eBioscience) was used as analyte at concentrations between 0.5 and 40 nM and kinetic data were collected at a flow rate of 30 μl min^−1^. For each sample, a 3-min association phase was followed by a 20-min dissociation phase. The chip was regenerated between consecutive samples with 2 pulses of 30 s with 10 mM glycine–HCl buffer pH 1.5. After double reference-subtraction, sensorgrams were analysed using the BiaEvaluation software (version 4.1). Kinetic parameters were fitted to a 1:1 Langmuir model.

### Mass spectrometry

Two μl of the same sample used in the crystallization experiments was diluted in 50 μl of 50 mM ammonium bicarbonate pH 7.5. The sample was subsequently digested with trypsin (Porcine, sequencing grade, Promega, Madison, WI) O/N at 37 °C using an enzyme to protein ratio 1:20. Formic acid was added to a final v/v concentration of 0.1% before the liquid chromatography mass spectrometry analysis. The digest was separated using an ultra performance Nano Acquity system coupled to a Synapt G1 mass spectrometer (Waters, Milford, MA). The peptides were separated on an HSS T3 (75 μm × 250 μm, 1.8 μm particles) column (Waters) with a gradient of 3–40% of buffer A (0.1% formic acid) and buffer B (100% acetonitrile with 0.1% formic acid). The spectra were acquired in Liquid-chromatography mass spectrometry in elevated energy mode (LCMS^E^) mode (alternating low and high collision energy) with mass range from 125 to 2,000 *m*/*z* using a collision energy ramp (10–40 V)^61^. The data were analysed using the Proteinlynx Global Server V2.5 platform (Waters) against UniProt database using C-mannosylation as a variable modification.

## Author contributions

J.D., S.L., E.L. and I.L. designed and developed the Alphabody scaffold. K.V.B. expressed and purified recombinant proteins. K.V. and Y.B. carried out ITC and structural studies of the IL-23:MA12 complex with contributions from SNS in structural analysis. S.D. carried out SPR measurements. E.L. carried out CD and stability experiments. P.H. supervised and executed the biopanning campaigns, ELISAs and splenocyte assays. K.S. carried out the cellular assays and supervised the *in vivo* studies. Y.W. and B.D. carried out mass spectrometry. I.L. and S.N.S. designed and supervised the study. S.N.S. wrote the manuscript with contributions from all authors.

## Additional information

**Accession codes:** Atomic coordinates and structure factors for the IL-23–MA12 complex have been deposited in the Protein Data Bank with accession codes 4OE8 and 4OG9.

**How to cite this article**: Desmet, J. *et al.* Structural basis of IL-23 antagonism by an Alphabody protein scaffold. *Nat. Commun.* 5:5237 doi: 10.1038/ncomms6237 (2014).

## Supplementary Material

Supplementary Information Supplementary Figures 1-4, Supplementary Tables 1-4.

## Figures and Tables

**Table 1:** Binding characteristics of developed Alphabodies as determined via ELISA, kinetic ELISA, phage-coated ELISA, splenocyte assays and cellular assays employing a DB cell line.

**Clone no.**	**Alphabody**	**ELISA**	**Kinetic ELISA**			**Phage-coated ELISA**	**Splenocyte assay**	**DB assay**
		***K***_**D**_ **(nM)**	***k***_**on**_ **(M**^**−1**^ **s**^**−1**^**)**	***k***_**off**_ **(s**^**−1**^**)**	***K***_**D**_ **(nM)**	***K***_**D**_ **(nM)**	***K***_**I**_ **(nM)**	***K***_**I**_ **(nM)**
1	Cl59	3.80	1.0 × 10^5^	7.0 × 10^−4^	7.00	3.00	69.0	ND
2	MA12	0.11	8.1 × 10^5^	2.1 × 10^−4^	0.26	0.08	0.13	0.35
3	MB23	0.17	1.5 × 10^6^	2.1 × 10^−4^	0.15	0.07	0.20	0.16
4	MB64	0.21	1.1 × 10^6^	3.3 × 10^−4^	0.30	0.09	0.18	0.46
5	MA5	0.10	4.5 × 10^6^	1.6 × 10^−4^	0.03	0.20	1.15	0.45
6	MA15	0.31	8.0 × 10^5^	7.2 × 10^−5^	0.09	0.12	0.48	0.29
7	MB9	0.27	1.9 × 10^6^	2.5 × 10^−4^	0.13	0.20	0.36	0.70
8	MA9	0.17	1.4 × 10^6^	3.5 × 10^−4^	0.25	0.20	0.46	0.24
9	MB38	0.20	1.0 × 10^6^	2.2 × 10^−4^	0.21	0.18	1.01	0.66
10	MB74	0.35	6.5 × 10^5^	1.6 × 10^−4^	0.25	0.47	0.90	1.80
11	MB67	0.25	1.6 × 10^6^	2.2 × 10^−4^	0.14	0.21	1.33	ND
12	MA14	1.80	2.0 × 10^5^	5.0 × 10^−4^	2.50	1.50	11.0	ND
13	MA23	0.29	4.5 × 10^5^	3.2 × 10^−4^	0.70	0.70	10.0	ND
14	MB43	0.20	1.3 × 10^6^	1.4 × 10^−4^	0.11	0.18	0.88	ND
15	MB76	1.80	2.5 × 10^5^	5.6 × 10^−4^	2.30	0.16	1.70	ND
16	MAcons	0.23	2.5 × 10^6^	3.2 × 10^−4^	0.13	ND	4.10	ND
17	MBcons	2.80	2.2 × 10^5^	5.9 × 10^−4^	2.70	ND	0.65	0.80
18	Cl59m	ND	ND	ND	ND	ND	22.0	1.73
19	59m_C2eQ	0.93	1.1 × 10^5^	4.3 × 10^−4^	3.90	ND	1.90	ND
20	59m_A3cA_A4cS_C2eQ	0.39	3.5 × 10^5^	2.6 × 10^−4^	0.75	ND	0.70	ND

ELISA, enzyme-linked immunosorbent assay; ND, not determined.

**Table 2:** Crystallographic data collection and refinement statistics.

	**Crystal form 1**	**Crystal form 2**
*Data collection*		
Space group	P12_1_1	P4_1_2_1_2
Unit cell dimensions		
*a*, *b*, *c* (Å)	56.96 56.74 100.07	57.85 57.85 366.48
*α β γ* (°)	90, 99.73, 90	90, 90, 90
Resolution (Å)	41–1.74 (1.85–1.74)	57–3.4 (3.5–3.4)
*R*_meas_ (%)	5.4 (63.6)	11.6 (92.1)
<*I*/σ(*I*)>	13.7 (1.8)	20.7 (5.2)
Completeness (%)	97.9 (91.1)	99.85 (100.0)
Redundancy	3.4 (3.4)	22.3 (23.4)
		
*Refinement*		
Resolution (Å)	40–1.7	57–3.4
No. of reflections	213960 (32767)	212910 (9551)
*R*_work_/*R*_free_	0.17/0.22 (0.27/0.33)	0.26/0.29 (0.34/0.38)
No. of atoms*		
Protein	4,295	4,060
Ligand/ion/glycan	35/2/61	−/−/60
Water	410	—
*B*-factors (Å^2^)*		
Protein	38.7	116.3
Ligand/ion/glycan	62.9/39.7/34.4	−/−/104.6
Water	41.6	—
R.m.s.d.		
Bond lengths (Å)	0.007	0.005
Bond angles (°)	1.04	1.1

r.m.s.d., root mean squared deviation.

Each of the reported data sets was obtained from one crystal.

^*^Hydrogens excluded.

Values in parentheses refer to the highest-resolution shell.
