# Supplementary material for: Structural basis of IL-23 antagonism by an Alphabody protein scaffold
Source: Nat Commun. 2014 Oct 30;5:5237. doi: 10.1038/ncomms6237 (PMC4220489; doi:10.1038/ncomms6237)
Supplement: Supplementary Information — Supplementary Figures 1-4, Supplementary Tables 1-4. [file ncomms6237-s1.pdf]

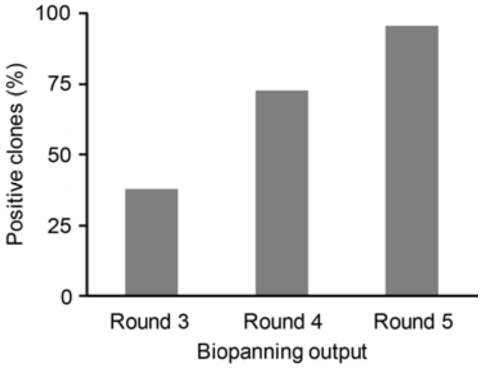

Supplementary Figure 1. Biopanning and clone enrichment of Alphabody binders against human IL-23. Positive clones in phage ELISA with optical density (OD) 3 times higher than background are shown for the output of rounds 3, 4 and 5.

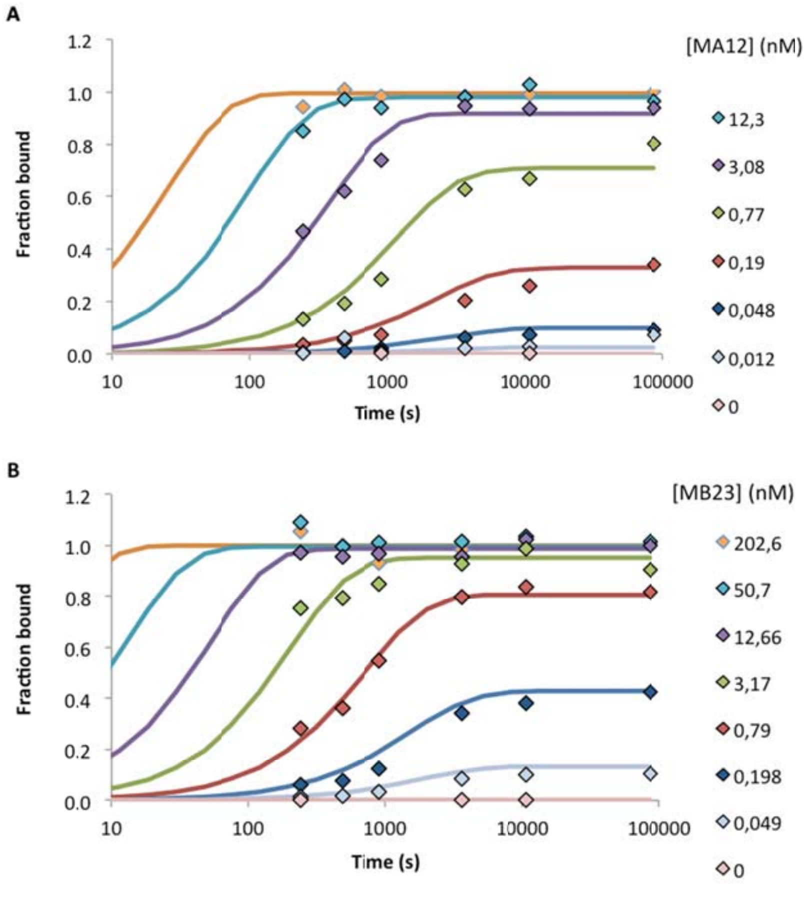

Supplementary Figure 2. Kinetic ELISA binding profiles for two representative affinity-matured Alphabodies, MA12 (a) and MB23 (b). The concentrations of MA12 and MB23 used for each binding curve are shown to the right of each set of binding curves.

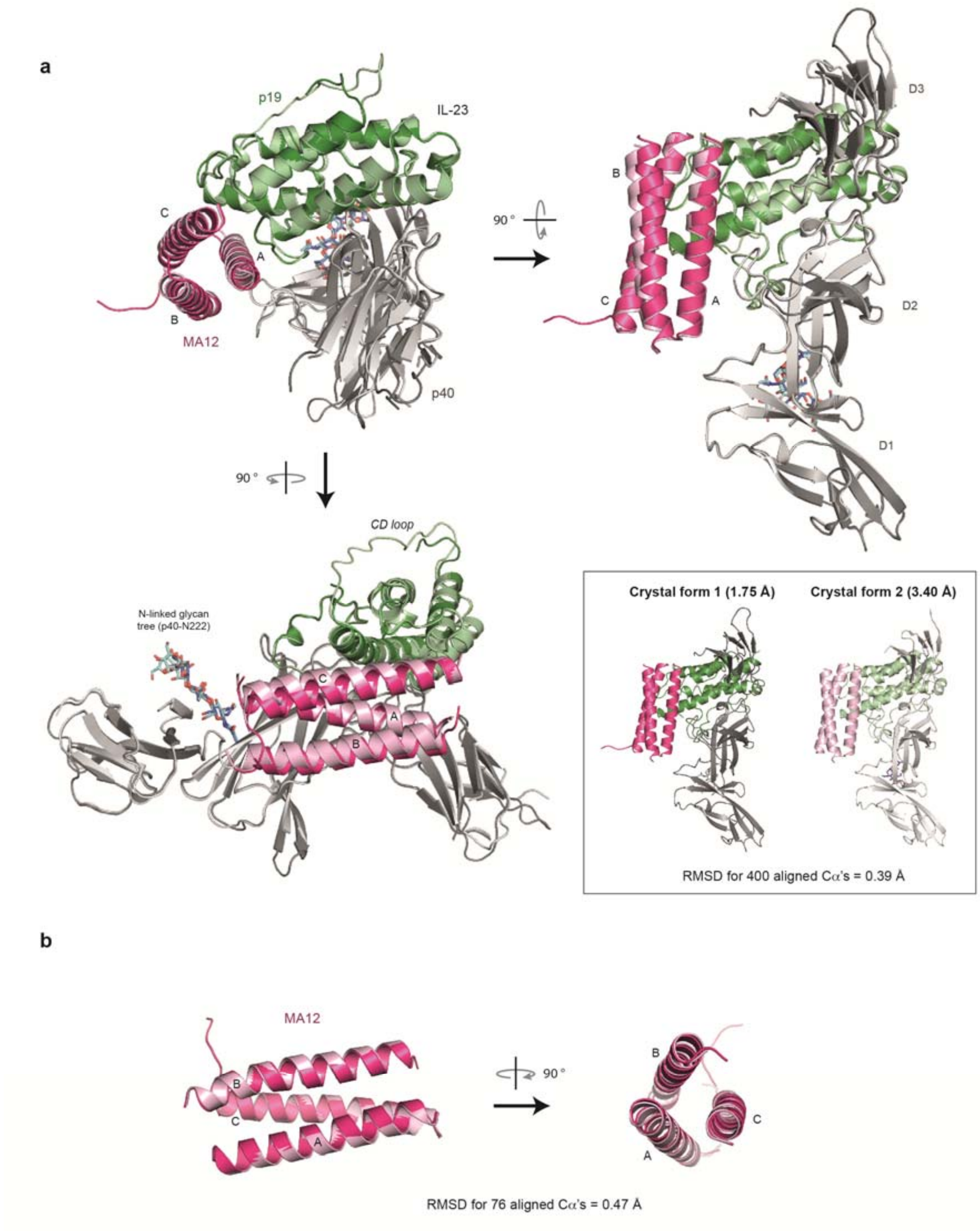

Supplementary Figure 3

a. Structural comparison between the two determined structures for the IL-23:MA12 complex. The overall RMSD between the two structures is 0.39 Å for 400 aligned Cα residues. **b.** Structural superposition of the MA12 Alphabody in crystal form 1 and 2. Both structures superimpose very well, indicating that the Alphabody fold remains largely invariant.

a

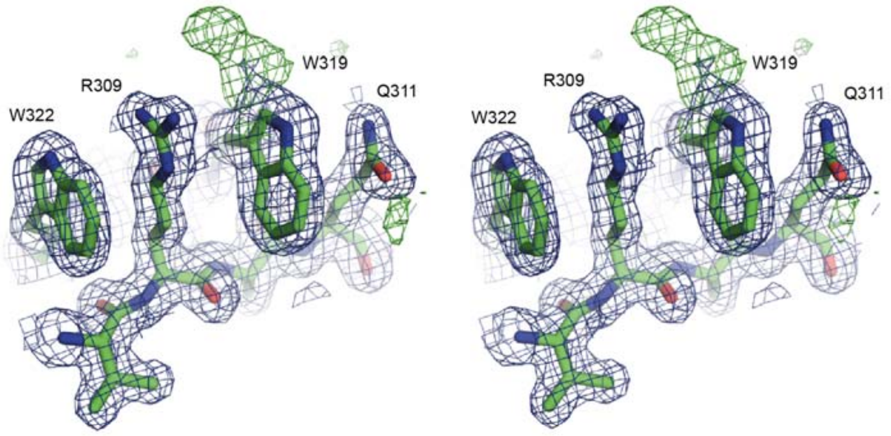

b

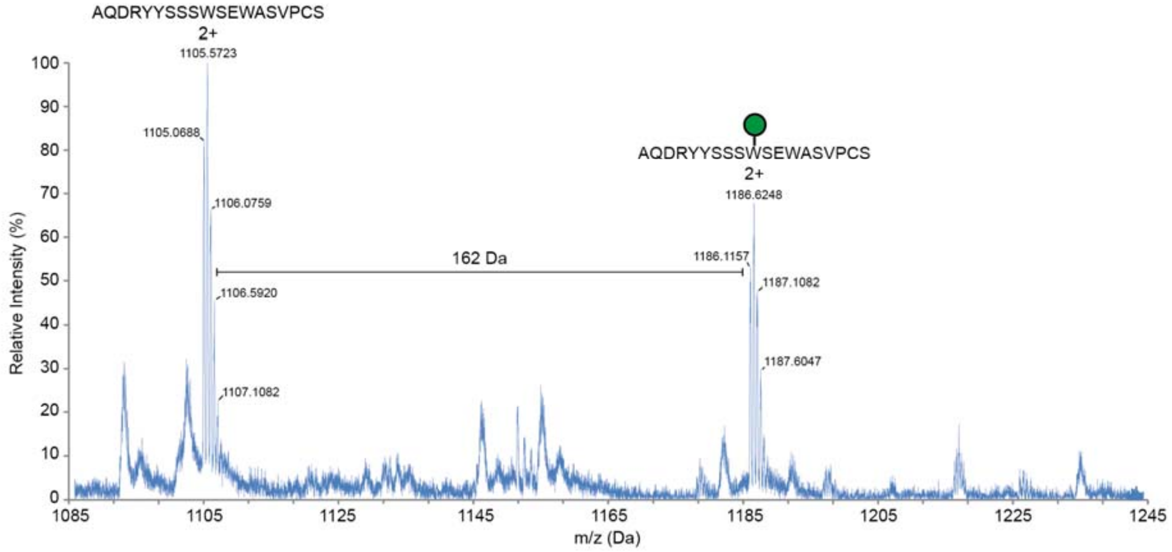

Precursor MH+ (Da)	z	Peptide MH+ (Da)	MH+ Error (ppm)	Sequence	Modifications	Intensity
2370.9822	2	2370.983	-0.3421	(R)AQDRYYSSSWSEWASVPCS(-)	C-Mannosyl W	25659
2208.9314	2	2208.9292	1.0361	(R)AQDRYYSSSWSEWASVPCS(-)		42111

Supplementary Figure 4. Characterization of C-mannosylation in human IL-23.
a. Stereo-view of difference electron density around W319, located in the WXXW motif of p40-D3, suggesting C-mannosylation of atom CD1 of the tryptophan side-chain. The blue mesh corresponds to difference electron density calculated with 2Fo-Fc;α_C Fourier coefficients and is contoured at 1σ r.m.s.d. The green mesh shows positive difference density calculated with 2Fo-Fc;α_C Fourier coefficients and is contoured at 3σ r.m.s.d. Residue numbering in the structure of human IL-23 reported herein reflects the sequence numbering of the protein in Uniprot. Thus, residue numbers in the p40 subunit of human IL-23 differ by 22 with respect to equivalent residues in PDB entries 3DUH, 3D85, 3D87, 3QWR and 4GRW (e.g. W319 in the p40 subunit of human IL-23 is equivalent to W297 in previously reported structures). **b.** A mass shift of 162 Da was observed in LC-MS after tryptic digest of the sample used for the crystallization experiments. This mass shift concurs with hexosylation of the peptide, most likely the C-mannosylation of W319 at position CD1 of the tryptophan side-chain.

Supplementary Table 1. Identification and sequence characteristics of Alphabody variants able to bind to human IL-23.

	hIL-23	"+mAB"	Inhibition	HELIX A(4)						HELIX C(4)				
clone	OD(1)	OD(2)	%(3)	1g	2c	2g	3c	3g	4c	2d	2e	3b	3e	4b
52	3.00	0.14	95.3	E	Q*	M	A	W	S	A	G	V	F	A
39	2.74	2.41	12.2	S	P	Q*	Q*	N	E	D	A	W	M	N
40	2.51	2.22	11.4	P	S	Q*	Q*	L	W	T	T	L	F	I
44	2.08	0.10	95.3	P	Q*	I	A	Y	R	W	K	I	Y	L
38	1.76	1.99	-13.5	Q*	T	Q*	M	I	A	S	G	T	F	F
63	1.33	0.83	37.6	P	R	Q*	R	G	K	K	S	Y	V	M
59	1.29	0.05	96.0	Q*	S	V	G	W	M	T	K	I	Y	M
72	1.05	0.72	31.7	G	G	Q*	K	G	M	Q*	S	V	F	W
45	0.67	0.47	29.9	P	S	R	R	Q*	M	N	A	T	F	I
33	0.45	0.07	85.2	Q*	T	I	S	Y	G	Y	Q*	L	Y	M

(1) Phage ELISA Optical Density (OD); (2) competition ELISA OD in the presence of neutralizing antibody B-Z23; (3) percentage inhibition by the B-Z23 antibody, derived from the previous two columns; (4) amino acids at the variable library positions as indicated below (for example, Helix A position 1g denotes the g-position in the first heptad of the A-helix; 'Q*' means an amber stop codon translated into glutamine (Q).

The consensus binding motif as derived from the B-Z23 inhibited clones 52, 44, 49, and 33 can be described as follows:

position A2g: aliphatic (M/I/V); A3c: small (A/G/S); A3g: aromatic (W/Y); C3b: aliphatic (V/I/L); C3e: aromatic (F/Y).

Amino acid position definitions in each helix are coded as follows:

-first character denotes the helix.

-second character specifies the relevant heptad repeat.

-third character identified the position in the heptad repeat.

e.g. A2g: Amino acid at position *g* in the second heptad of helix A.

Supplementary Table 2. Distribution of amino acids for each variable position in the maturation library 'matLib'. The varied positions are indicated in the first column (code: helix-heptad-position). The numbers are expressed as percentages. The column headed by an asterisk indicates a stop codon.
Amino acid position definitions are coded as follows:
-first character denotes the helix.
-second character specifies the relevant heptad repeat.
-third character identified the position in the heptad repeat.
e.g. A1b: Amino acid at position *b* in the first heptad of helix A.

	G	A	P	C	S	T	V	I	L	M	F	Y	W	H	N	Q	D	E	K	R	*
A1b																48		48	4		
A1c																48		48	4		
A1f		3				13												13	71		
A1g																48		48	4		
A2b		71				13												13	3		
A2c			1		17	3								8	14	8			14	35	
A2f		8					4											88			
A2g							32	32	2	32	2										
A3c	38	30			18	14															
A3g												50	50								
A4c	7	15			15	23	7		7	11			7							11	
C2b		88			4	8															
C2e					4			1	2	2				8	8	23			25	28	
C2f	6	10	3		5	12						1		2	8	5	7	14	17	7	3
C3b							32	32	33		3										
C3e											52	48									
C4b	4	3	3	3	9	6	5	6	10	6	4	3	3	3	6	3	2	2	6	10	3
C4e	4	3	3	3	9	6	5	6	10	6	4	3	3	3	6	3	2	2	6	10	3
C4f	7	6	4	3	8	6	8	4	9	4	3	3	3	3	4	3	4	4	4	8	3

Supplementary Table 3. Sequence characteristics of affinity-matured Alphabodies against human IL-23. Cl59 is provided for comparison purposes.
Amino acid position definitions in each helix are coded as follows:
-first character specifies the relevant heptad repeat.
-second character identified the position in the heptad repeat.
e.g. 1b: Amino acid at position *b* in the first heptad.
L1 and L2 correspond to the interhelix linker segments as defined in Figure 1. L8 and L16 denote the linker lengths in terms of the number of amino acids involved.

		HELIX A												HELIX B		HELIX C							
#	Alphabody	1b	1c	1f	1g	2b	2c	2f	2g	3c	3g	4c	L1	2f	L2	2b	2e	2f	3b	3e	4b	4e	4f
1	**Cl59**	E	E	K	Q	A	S	E	V	G	W	M	**L16**	-	**L16**	T	K	E	I	Y	M	T	P
2	**MA12**	Q	E	K	E	A	Q	A	V	G	Y	T	**L16**	C	**L16**	A	Q	E	L	Y	M	V	T
3	**MB23**	E	Q	K	E	T	T	E	V	A	Y	T	**L8**	C	**L8**	A	Q	E	V	Y	M	A	S
4	**MB64**	Q	Q	K	E	T	Q	A	V	G	Y	R	**L16**	C	**L8**	A	Q	G	V	Y	M	A	T
5	**MA5**	K	Q	K	E	A	Q	E	V	A	W	R	**L8**	C	**L16**	A	Q	D	V	Y	M	S	S
6	**MA15**	Q	E	K	E	A	N	A	V	G	Y	T	**L16**	C	**L8**	T	Q	A	I	Y	M	P	R
7	**MB9**	Q	Q	K	E	T	N	E	V	A	W	T	**L8**	C	**L16**	A	Q	N	L	Y	M	G	S
8	**MA9**	Q	Q	K	E	T	T	A	V	A	W	T	**L16**	C	**L8**	A	Q	G	I	Y	M	K	D
9	**MB38**	Q	E	K	E	K	Q	E	V	G	Y	G	**L8**	C	**L8**	A	Q	N	L	Y	M	P	Q
10	**MB74**	Q	Q	K	E	A	T	E	V	G	Y	R	**L16**	C	**L8**	A	Q	G	L	Y	M	A	I
11	**MB67**	Q	K	K	E	T	N	E	V	A	Y	T	**L8**	C	**L16**	A	Q	R	L	Y	M	S	T
12	**MA14**	E	Q	T	E	A	S	E	V	G	Y	S	**L8**	C	**L8**	A	Q	N	V	Y	M	G	G
13	**MA23**	Q	Q	K	E	A	N	E	V	A	W	L	**L8**	C	**L8**	A	Q	E	V	Y	M	P	S
14	**MB43**	E	E	K	E	K	N	E	V	A	Y	T	**L8**	C	**L16**	A	Q	D	I	Y	M	T	R
15	**MB76**	Q	Q	K	E	T	Q	E	V	A	Y	R	**L8**	C	**L8**	A	Q	G	L	Y	M	S	L
16	**MAcons**	Q	Q	K	E	A	Q	E	V	A	W	S	**L16**	C	**L8**	A	Q	D	V	Y	M	S	G
17	**MBcons**	Q	Q	K	E	K	Q	E	V	A	Y	R	**L8**	C	**L8**	A	Q	D	L	Y	M	A	Q
18	**Cl59m**	Q	Q	K	E	A	Q	E	V	G	W	M	**L16**	-	**L16**	T	K	E	I	Y	M	T	P
19	**59m_C2eQ**	Q	Q	K	E	A	Q	E	V	G	W	M	**L16**	C	**L16**	T	Q	E	I	Y	M	T	P
20	**59m_A3cA_A4cS_C2eQ**	Q	Q	K	E	A	Q	E	V	A	W	S	**L16**	C	**L16**	T	Q	E	I	Y	M	T	P

Supplementary Table 4. Interactions between Alphabody MA12 and human IL-23/p19. Residue numbering in the structure of human IL-23 reported herein reflects the sequence numbering of the protein in Uniprot. Thus, residue numbers in the p19 subunit of human IL-23 differ by 19 with respect to equivalent residues in PDB entries 3DUH, 3D85, 3D87, 3QWR and 4GRW (e.g. His48 in the p19 subunit of human IL-23 is equivalent to His29 in previously reported structures). Each residue in MA12 is labeled according to its occurrence at a variable (v) versus a core (c) library position.

Hydrogen bonds and salt-bridge interactions

Alphabody	IL-23 p19	Distance (Å)
(v) Tyr110 OH	His48 Nε2	2.73
(v) Tyr110 OH	Arg162 Nη1	3.02
(v) Tyr24 OH	Ala47 O	3.25
(c) Lys111 Nζ	Glu112 Oε2	2.92

Water-mediated interactions

Alphabody	Distance	Water	Distance (Å)	IL-23 p19
(v) Ala16 O	2.8	Wat-43	3.3	Asp55 Oδ2
(v) Thr118 Oγ1	2.8	Wat-57	3.0	Leu116 N
(v) Thr27 Oγ1	2.9	Wat-176	2.8	Ser46 O
(v) Gly20 O	2.8	Wat-201	2.8	Leu50 N
(v) Tyr24 OH	2.6	Wat-396	2.9	Ser46 O
(v) Gln13 Oε1	2.0	Wat-401	3.0	Trp156 Nε2

van der Waals interactions

Alphabody	IL-23 p19
(v) Gln13	Leu56, Leu160
(c) Ile14	Trp156
(v) Ala16	Leu56, Asp55, Val51
(v) Val17	Phe163, Leu160, Leu50
(c) Ile21	Pro49
(c) Tyr24	Pro49, His48, Ser46
(v) Gln103	Trp156
(c) Ile106	Trp156
(v) Leu107	Trp156, Leu159, Pro155, Pro113
(v) Tyr110	Pro113, His48, Leu159
(c) Lys111	Pro113
(v) Met114	Leu115, Pro49
(v) Val117	Pro120
(v) Thr118	Ser119, Asp118, Leu116
